# Low levels of vaccine coverage and immunity against hepatitis B virus in children with hematological malignancies in Brazil

**DOI:** 10.1016/j.bjid.2025.104575

**Published:** 2025-08-28

**Authors:** Vitória Machado Krüger, Alexandre Lemos da Silva, Luciano Zubaran Goldani

**Affiliations:** a Universidade Federal do Rio Grande do Sul, Programa de Pós-Graduação em Ciências Médicas, Porto Alegre, RS, Brazil; b Secretaria da Saúde do Estado do Rio Grande do Sul, Centro Estadual de Vigilância em Saúde, Porto Alegre, RS, Brazil; c Universidade Federal de Ciências da Saúde de Porto Alegre, Porto Alegre, RS, Brazil; d Universidade Federal do Rio Grande do Sul, Departamento de Medicina Interna, Porto Alegre, RS, Brazil

**Keywords:** Hepatitis B, Vaccines, Antibodies, Pediatric, Hematological malignancies

## Abstract

Hepatitis B remains a global health concern due its high prevalence and association with chronic liver disease. Although vaccination is safe and effective in immunocompetent individuals, patients with hematological malignancies often exhibit immune dysfunction and reduced vaccine responses, increasing their susceptibility to vaccine-preventable infections. This study aimed to assess the hepatitis B vaccination status and immunoprotection in pediatric oncology patients treated at a tertiary public hospital in southern Brazil. A retrospective, single-center study was conducted with patients aged 0 to 15 years undergoing treatment for hematological malignancies between 2020 and 2022. Clinical and demographic data were obtained from electronic medical records, including vaccination history and hepatitis B serology profile. Vaccination status was verified through the National Immunization Program Information System. A total of 101 patients were evaluated, comprising 58 (57.4 %) males and 43 (42.6 %) females. The predominant diagnosis was acute lymphoblastic leukemia, reported in 67 cases (66.3 %). All patients underwent chemotherapy protocols, and 28 (27.7 %) died during the study period. Serological data for hepatitis B were available for 66 patients (65.3 %), with the highest proportions of missing data for anti-HBs (25.7 %) and total anti-HBc (13.9 %). None tested positive for HBsAg, 2 (2.3 %) were positive for total anti-HBc and 33 (44.0 %) for anti-HBs. Regarding vaccination status, 62 (61.4 %) had completed the hepatitis B vaccine series, 12 (11.9 %) had incomplete schedules, and 27 (26.7 %) had no records available. Only 10 patients (13.5 %) received additional vaccination after oncological diagnosis. Among those with a complete vaccination schedule, 27 (55.1 %) did not develop protective antibodies. These findings demonstrate low level of immunoprotection and suboptimal vaccine coverage against hepatitis B in this population. Optimizing vaccination protocols and monitoring strategies are essential to ensure adequate immunization against hepatitis B and other preventable diseases in immunosuppressed pediatric patients receiving care in Brazil.

## Introduction

Hepatitis B Virus Infection (HBV) is a major global health concern and a leading cause of chronic liver disease, significantly contributing to the burden of liver-related morbidity and mortality worldwide. It is estimated that 254 million people are infected with the vírus, and approximately 1.1 million deaths were attributed to HBV-related causes in 2022.^1^ While most immunocompetent adults experience an asymptomatic, self-limiting infection (with < 5 % progressing to chronicity), children and immunosuppressed individuals face a higher risk of chronic infection ‒ reaching up to 90 % in infected newborns.^2^^,^^3^ Among chronically infected individuals, 15 %–25 % die from complications such as cirrhosis or hepatocellular carcinoma.^4^

Vaccination remains the primary method of prevention. Administering the vaccine at birth and during childhood is a key strategy for the global elimination of the disease and has been highly effective in reducing the incidence of new infections.^5^ The vaccine is recommended for newborns, pregnant women, unvaccinated adults, and individuals at increase risk for HBV.^6^ The World Health Organization recommends the first dose be administered within 12 to 24 hours after birth, followed by 2 or 3 additional doses to complete the primary immunization series.^7^ In immunocompetent individuals, completion of the vaccination schedule induces a protective immune response (anti-HBs ≥10 mIU/mL), achieved in approximately 95 % of vaccinated children. Given this high efficacy, routine post-vaccination serological testing is not recommended for the general population. However, in immunosupressed patients, including those with malignancies or candidates for immunosuppressive treatments, serological monitoring should be conducted.^8^, ^9^, ^10^ This practice is important because vaccine-induced immunity may be suboptimal in these groups, and alternative immunization strategies, including higher antigen doses, additional booster doses, or modified schedules, are often required to ensure adequate protection.^11^ New generations of vaccines and double-dose of recombinant hepatitis B vaccine are options for patients who are non-responder to standard HBV vaccination.^12^^,^^13^ In Brazil, the vaccination of oncology patients and individuals with other clinical conditions is conducted through the Reference Centers for Special Immunobiologicals, based on medical prescriptions and established clinical protocols.^14^

The assessment of immunoprotection is particularly relevant in groups at high risk of exposure to hepatitis B, such as individuals with hematological malignancies.^15^, ^16^, ^17^ These malignancies represent one of the most prevalent types of childhood cancers, with leukemia alone comprising 30 % of pediatric cancer cases.^18^ A characteristic of these conditions is their potential to compromise immune function, both through disease's inherent mechanisms and as a result of aggressive treatments, including chemotherapy, hematopoietic stem cell transplantation, and repeated blood transfusions.^19^^,^^20^ Consequently, children with hematological malignancies are at an increased risk of infections potentially preventable by immunization, including a higher vulnerability to HBV and progression to chronic disease.^21^ For these patients, in addition to assessing vaccination status, clinical guidelines recommend hepatitis B screening to detect occult infection and prevent viral reactivation.^11^ Reactivation is defined by the reappearance or increase in viral DNA levels in the serum of previously exposed individuals, typically associated with immune system suppression. It is linked to adverse clinical outcomes, ranging from self-limiting hepatitis to fulminant liver failure and death.^22^^,^^23^ Given the risk faced by this population, the present study aims to evaluate the vaccination status and immunoprotection profile of hepatitis B in children with hematological malignancies in a tertiary public hospital in Brazil, contributing to disease burden monitoring and supporting the development of alternative prevention strategies.

## Material and methods

### Study design, patients and settings

This was a single-center, observational, retrospective, and descriptive study conducted at the Pediatric Oncology and Hematology service of the Hospital de Clínicas de Porto Alegre ‒ a 700-bed tertiary care hospital located in southern Brazil. These services provide specialized care to children and adolescents from birth up to 18-years of age and are recognized as national referral centers for pediatric and adolescent cancer treatment in the country. The study population comprised patients undergoing treatment for hematological malignancies between January 2020 and December 2022. Inclusion criteria were patients of both sexes, aged ≤15 years, with a confirmed diagnosis of hematological malignancies, whose legal guardians had provided consent to share contact information with researchers affiliated with the institution. Patients diagnosed with HIV, other immunodeficiencies, or without any available hepatitis B serology results were excluded. Eligible participants were identified through the hospital’s electronic database using International Classification of Diseases (ICD) codes corresponding to hematological malignancies, along with the defined inclusion and exclusion criteria. This process resulted in a total of 101 patients.

### Variables analyzed

Data were retrospectively collected through the review of electronic medical records. The extracted variables included: full name, date of birth, maternal name, age, sex, race/ethnicity, place of birth, clinical diagnosis, therapeutic interventions (chemotherapy, radiotherapy, immunotherapy, and/or hematopoietic stem cell transplantation), vaccination history, HBV serological profile and mortality status. The HBV serological profile was determined based on the results of screening tests performed during immunosuppressive therapy. The most recent available results for Hepatitis B surface Antigen (HBsAg), total Hepatitis B core antibody (total anti-HBc), and Hepatitis B surface antibody (anti-HBs) were considered for analysis. All serological tests were conducted by the institution’s diagnostic laboratory using the Microparticle Chemiluminescence Immunoassay (CMIA) methodology. Patients were classified as either positive or negative for each serological marker. Anti-HBs titers ≥ 10 mIU/mL were interpreted as indicative of seroprotection. Cases with no available serological data were categorized as “not performed”. Vaccination status was verified through the National Immunization Program Information System (SI-PNI), in accordance with the national basic immunization schedule. A complete hepatitis B vaccination schedule was defined as the documentation of three or more doses, incomplete when less than three doses were recorded, and unregistered when no doses were available in the system. Vaccine doses administered after the oncological diagnosis in patients with a prior vaccination record were considered additional vaccinations.

### Statistical analysis

Data analysis was conducted using the Statistical Package for the Social Sciences (SPSS), version 22.0 (IBM Corp., Armonk, NY, USA). Descriptive statistics were applied, including absolute and relative frequencies for categorical variables, as well as measures of central tendency and dispersion for continuous variables – mean and standard deviation.

### Ethical aspects

This study was approved by the ethics committee of the involved institutions (CAAE 67.073.723.8.0000.5327 and 67.073.723.8.3001.5312). Informed consent was obtained from each patient or their legal representative.

## Results

### Patients’ characteristics

As shown in Table 1, 101 patients diagnosed with hematologic malignancies, including 58 (57.4 %) males and 43 (42.6 %) females were enrolled in this study. Ages ranged from 0 to 15 years, with a median age of 6 ± 4 years at the time of diagnosis. Acute lymphoblastic leukemia was the most prevalent malignancy, accounting for 67 (66.3 %) cases. Non-Hodgkin’s lymphoma and Hodgkin lymphoma were identified in 14 (13.9 %) and 2 (2.0 %) patients, respectively. All patients received chemotherapy-based treatment protocols. Chemotherapy was administered as monotherapy in 59 patients (58.4 %), while the remaining 42 patients (41.6 %) received chemotherapy in combination with other therapeutic modalities. Among these, 9 patients (8.9 %) received chemotherapy and radiotherapy, 3 (3.0 %) chemotherapy and immunotherapy, 8 (7.9 %) chemotherapy followed by hematopoietic stem cell transplantation (HSCT), 13 (12.9 %) chemotherapy, radiotherapy and HSCT, and 9 (8.9 %) chemotherapy combined with radiotherapy, immunotherapy and HSCT. During the study period, 28 patients (27.7 %) died.

**Table 1:** Characteristics of patients with hematological malignancies.

Variables ‒ n ( %)	(*n* = 101 / 100 %)
**Sex**	
Male	58 (57.4 %)
Female	43 (42.6 %)
**Color**	
White	83 (82.2 %)
Black	15 (14.8 %)
Brown	3 (3.0 %)
**Age, years**	
0 ‒ 4	38 (37.6 %)
5 ‒ 9	38 (37.6 %)
10 ‒ 15	25 (24.8 %)
**Diagnosis**	
Acute Lymphoblastic Leukemia	67 (66.3 %)
Acute Myeloid Leukemia	15 (14.8 %)
Juvenile Myelomonocytic Leukemia	3 (3.0 %)
Hodgkin's Lymphoma	2 (2.0 %)
Non-Hodgkin's Lymphoma	14 (13.9 %)
**Treatment**	
Chemotherapy	59 (58.4 %)
Chemotherapy + Radiotherapy	9 (8.9 %)
Chemotherapy + Immunotherapy	3 (3.0 %)
Chemotherapy + HSCT	8 (7.9 %)
Chemotherapy + Radiotherapy + HSCT	13 (12.9 %)
Chemotherapy + Radiotherapy + Immunotherapy + HSCT	9 (8.9 %)
**Outcome**	
Death	28 (27.7 %)

HSCT, Hematopoietic Stem Cell Transplantation.

### HBV serology

Complete hepatitis B serological testing (HBsAg, total anti-HBc, and anti-HBs) was performed in 66 patients (65.3 %). The highest proportions of missing data were observed for anti-HBs (25.7 %) and total anti-HBc (13.9 %) tests. Only one patient lacked HBsAg results. Among those with available serological data, all tested negative for HBsAg, indicating the absence of active infection. However, 2 (2.3 %) patients showed markers of prior exposure to the virus, as evidenced by positive total anti-HBc results. Additionally, 33 patients (44.0 %) tested positive for anti-HBs, indicating protective antibodies titers against hepatitis B (Table 2).

**Table 2:** Hepatitis B serology results.

Variables	Positive	Negative	Total (*n* = 100 %)
HbsAg	0	100 (100 %)	100
Total anti-HBc	2 (2.3 %)	85 (97.7 %)	87
Anti-HBs	33 (44.0 %)	42 (56.0 %)	75

### Vaccination status and correlation with the serological profile

**Fig. 1:**
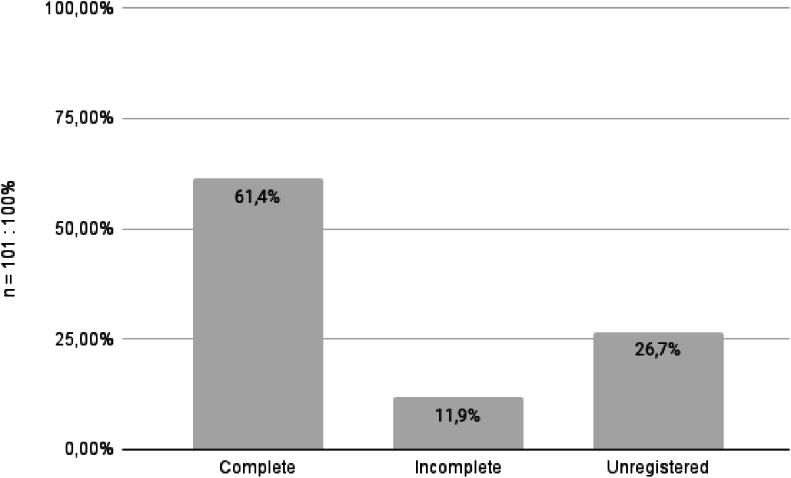
**Hepatites B vaccination status.** The figure illustrates the distribution of hepatitis B vaccination status among patients — categorized as complete (≥ 3 doses), incomplete (< 3 doses), or unregistered (no recorded doses).

Vaccination records were available for 74 patients (73.3 %) through the National Immunization Program Information System (SI-PNI). Vaccination history was assessed through the institution’s medical records for only 14 patients (13.9 %). Among the 101 patients evaluated, 62 (61.4 %) had completed the full hepatitis B vaccination schedule, whereas 12 (11.9 %) had incomplete schedule ‒ 5 patients had received only one dose, and 7 had received two doses. A total of 27 patients (26.7 %) had no vaccination records in the system and were classified as unvaccinated for hepatitis B (Fig. 1). For those with available vaccination data, 10 patients (13.5 %) received additional vaccine doses following their oncological diagnosis.

Regardless of vaccination status, no cases of active hepatitis B infection were detected. However, the two cases of past infection identified had no documented vaccination records. The patients, aged between 5 and 9 years, had been diagnosed with acute lymphoblastic leukemia. Both underwent chemotherapy protocols for oncological treatment and subsequently died. In terms of anti-HBs status, among patients with a complete vaccination schedule, only 22 (44.9 %) displayed protective titers. Furthermore, 8 patients without documented vaccination records also tested positive for anti-HBs. Of these, 2 had evidence of prior exposure to the virus, suggesting that the remaining patients may have been vaccinated at some point, but their vaccination data were not properly recorded (Table 3).

**Table 3:** Vaccination status and serological profile for hepatitis B.

	Vaccination statu	Serological profile				
		Positive		Negative		Total
		n	%	n	%	(*n* = 100 %)
**HbsAg**	Complete	0	0 %	61	100 %	61
	Incomplete	0	0 %	12	100 %	12
	Unregistered	0	0 %	27	100 %	27
**Total anti-HBc**	Complete	0	0 %	53	100 %	53
	Incomplete	0	0 %	11	100 %	11
	Unregistered	2	8.7 %	21	91.3 %	23
**Anti-HBs**	Complete	22	44.9 %	27	55.1 %	49
	Incomplete	3	42.9 %	4	57.1 %	7
	Unregistered	8	42.1 %	11	57.9 %	19

## Discussion

The implementation of hepatitis B vaccination programs and the enhancement of screening efforts have significantly reduced the prevalence of the virus. Globally, 115 countries have introduced universal hepatitis B birth-dose vaccination, and in 2022, approximately 45 % of infants received the vaccine.^1^ In the United States (US), coverage reached 81.5 % for the birth dose and 92.1 % for ≥3 doses among children born during 2019–2020.^24^ In Brazil, the National Immunization Program (PNI) is responsible for the provision and promotion of free vaccines and has achieved high immunization coverage, contributing to a notable decline in vaccine-preventable diseases in the last decades. In 2022, coverage rates for the birth dose and the third dose in the country were 82.76 % and 77.24 %, respectively ‒ both remaining below the targets established by the PNI.^25^

Despite the efforts of immunization programs, it remains a concern for cancer patients. Intensive therapies used in these individuals suppress the immune system, increasing their susceptibility to infections.^21^ For this population, clinical guidelines recommend serological screening during treatment and antiviral prophylaxis.^8^, ^9^, ^10^ Most studies conducted worldwide have focused on the burden of hepatitis B in the general population. This study, however, targets high-risk groups. Previous research has demonstrated a high prevalence of hepatitis B virus infection among individuals with hematological malignancies, particularly in countries with high endemicity.^15^^,^^26^^,^^27^ In Brazil, there is limited data addressing HBV prevalence and immunization status in oncological and pediatric populations. Although no cases of active infection were identified, low vaccination coverage and seropositivity rates for anti-HBs antibodies are concerning in patients with hematological malignancies. Notably, 56 % of the analyzed cases lacked protective immunity against HBV. Similar findings have been reported in other studies. Kebudi et al. found an anti-HBs seropositivity rate of 42 % among children undergoing oncological treatment in Turkey.^28^ Anafy et al. reported an even lower rate of 34 % in pediatric patients treated for acute lymphoblastic leukemia.^29^ In US, a retrospective study assessing antibody titers for tetanus, varicella, measles, mumps, rubella, hepatitis B, and polio in pediatric sarcoma patients found that 64 % had negative antibody titers for HBV.^30^

Oncological patients often exhibit a reduced response to vaccines. The use of aggressive and prolonged treatments disrupts the balance between the host's immune system and viral replication, suppressing normal immunological responses and affecting both cellular and humoral immunity. As a result, there is a decrease or loss of protective levels from previously administered vaccines.^31^^,^^32^ In the present study, 55.1 % of children who had previously completed the full vaccination schedule lacked protective antibodies. This finding emphasizes the importance of serological monitoring, which is essential for disease screening and assessment of the need for immunization. According to our data, a considerable number of patients did not perform all monitoring serological tests as recommended by clinical protocols. The absence of these information compromises patient management, limiting the capacity to make informed clinical decisions and to detect occult infections ‒ factors that may adversely affect patient outcomes.

Additionally, gaps were identified in the documentation of vaccination history within medical records, as few patients had this information recorded. Evaluation of vaccination history is essential to ensure that patients are appropriately referred for immunization updates. In our study, 27.6 % of patients had no recorded vaccine doses in the SI-PNI, raising concerns about the absence of vaccination and the reliability of the information system. The SI-PNI was implemented to improve the registration and monitoring of immunization data in Brazil. All administered vaccines must be recorded in the system to support the collection of detailed and accurate information about vaccinated individuals. Despite progress in its implementation, several challenges persist related to system operation and data completeness.^33^, ^34^, ^35^ Moraes et al. assessed the concordance between vaccination cards and the SI-PNI for 4.050 children and found that 11 % had no records in the system.^36^ In our findings, the presence of HBV antibodies in patients without vaccination records and with no prior history of viral exposure suggests potential failures in the registration process.

In addition to routine immunization, cancer patients require specific recommendations for vaccination and evaluation of immunoprotection. Studies have shown that seronegative oncology patients who underwent revaccination or received at least one booster dose of the recombinant hepatitis B vaccine were able to develop protective antibody levels.^21^^,^^37^ Other studies reported that alternative accelerated vaccination schedules are feasible for achieving a serologic response during chemotherapy or under immunosuppressive conditions.^20^ In this context, our study evaluated hepatitis B vaccination coverage after oncological diagnosis and identified a low number of recorded vaccinations. In the state of Rio Grande do Sul, Brazil, two reference centers provide vaccination for individuals with special clinical conditions. Although these centers play a crucial role in immunization efforts, limitations such as inadequate physical infrastructure and insufficient human resources can directly affect the population's access to immunobiologicals.^12^ Other factors identified in our study ‒ including incomplete documentation of vaccination histories and the lack of serological monitoring data ‒ also contribute to reduced vaccination coverage.^38^

This study has some limitations, including a small sample size from a single-center institution. As a retrospective analysis, hepatitis B serology results were unavailable for some patients, preventing the assessment of serological status before and after treatment. Moreover, challenges related to SI-PNI data completeness may impact the recording of vaccine doses, potentially affecting the analysis of vaccination coverage. Despite these limitations, our findings contribute to the understanding of HBV seroepidemiology in pediatric oncology patients, reinforcing the importance of serological monitoring and vaccination status evaluation for vaccine-preventable diseases in immunosuppressed patients.

## Conclusion

The low rate of immunoprotection and the lack of vaccination records for hepatitis B highlight gaps in vaccination coverage and increased susceptibility within this population. Continued efforts to enhance screening programs and optimize vaccination protocols are essential to reducing the risk of HBV transmission and its associated complications.

## Funding

This study was not supported by any funding agency.

## Conflicts of interest

The authors declare no conflicts of interest.
